# A Tool for Computation of Changes in Na^+^, K^+^, Cl^−^ Channels and Transporters Due to Apoptosis by Data on Cell Ion and Water Content Alteration

**DOI:** 10.3389/fcell.2019.00058

**Published:** 2019-04-17

**Authors:** Valentina E. Yurinskaya, Igor A. Vereninov, Alexey A. Vereninov

**Affiliations:** ^1^Laboratory of Cell Physiology, Institute of Cytology, Russian Academy of Sciences, St-Petersburg, Russia; ^2^Peter the Great St-Petersburg Polytechnic University, St-Petersburg, Russia

**Keywords:** apoptosis, monovalent ions, channel parameters computation, ion transport, cell water, apoptotic volume decrease

## Abstract

Monovalent ions are involved in a vast array of cellular processes. Their movement across the cell membrane is regulated by numerous channels and transporters. Identification of the pathways responsible for redistribution of ions and cell water in living cells is hampered by their strong interdependence. This difficulty can be overcome by computational analysis of the whole cell flux balance. Our previous computational studies were concerned with monovalent ion fluxes in cells under the conditions of balanced ion distribution or during transition processes after stopping the Na^+^/K^+^ pump. Here we analyze a more complex case—redistribution of ions during cell apoptosis when the parameters keep changing during the process. New experimental data for staurosporine-induced apoptosis of human lymphoma cells U937 have been obtained: the time course of changes in cellular K^+^, Na^+^, Cl^−^, and water content, as well as Rb^+^ fluxes as a marker of the Na/K pump activity. Using a newly developed computational tool, we found that alteration of ion and water balance was associated with a 55% decrease in the Na^+^/K^+^-ATPase rate coefficient over a 4-h period, with a time-dependent increase in potassium channel permeability, and a decrease in sodium channel permeability. The early decrease in [Cl^−^]_i_ and cell volume were associated with an ~5-fold increase in chloride channel permeability. The developed approach and the presented executable file can be used to identify the channels and transporters responsible for alterations of cell ion and water balance not only during apoptosis but in other physiological scenarios.

## Introduction

A characteristic feature of apoptosis, one of the basic genetically encoded cell death mechanisms, in contrast to accidental death, is that it is not associated with cell swelling or plasma membrane rupture (Galluzzi et al., 2018). The apoptotic volume decrease (AVD) is a common but not mandatory symptom of the cell apoptosis (Maeno et al., 2000, 2006; Okada et al., 2001; Yurinskaya et al., 2005a,b; Bortner and Cidlowski, 2007). Cell swelling in apoptosis is prevented by the specific alteration of the monovalent ion balance in apoptotic cells, as the monovalent ions are major cell water regulators. It is believed that monovalent ions play an important role in apoptosis (Lang et al., 2005; Lang and Hoffmann, 2012; Bortner and Cidlowski, 2014; Hoffmann et al., 2015; Kondratskyi et al., 2015; Jentsch, 2016; Wanitchakool et al., 2016). However, this opinion is based mostly on the fact that ion channels and transporters are altered somehow during apoptosis and that their pharmacological or genetic modification has an effect on apoptosis. The mechanism of specific apoptotic alteration of cell ion and water balance has gotten much less attention than the molecular identity of channels and transporters involved in apoptosis. The mechanistic studies are hampered by the interdependence between ion fluxes via the numerous channels and transporters in the plasma membrane. This difficulty can be overcome by the computational analysis of whole-cell ion flux balance, which has been developed for normal cells (Jakobsson, 1980; Lew and Bookchin, 1986; Lew et al., 1991; Terashima et al., 2006; Vereninov et al., 2014, 2016). However, no successful analyses have been done on apoptotic cells. We have studied the relationships between alterations of the Na^+^/K^+^ pump or K^+^, Na^+^, and Cl^−^ channels and transporters and the apoptotic alteration of the entire cell water and ion balance in U937 cells treated with staurosporine (STS) and etoposide (Yurinskaya et al., 2005a,b, 2011). However, we lacked the necessary experimental data and a proper programme code for computation of transient processes in cell ion homeostasis and analyzed only apoptotic cells at a single time point, 4 h. Here, we studied ionic events during apoptosis development from 30 min to 4 h. The background data included K^+^, Na^+^, Cl^−^, and water contents and ouabain-sensitive and -resistant Rb^+^ influx in U937 cells that were induced to undergo apoptosis by STS. An original algorithm of the numerical solution of the cell monovalent ion flux balance equations and the programme code were developed, which allowed us to account for the continuous changes in the Na^+^/K^+^ pump activity. To our knowledge, this is the first attempt to study the dynamics of the alteration of K^+^, Na^+^, Cl^−^, and water contents during apoptosis. The approach developed to study STS-induced apoptosis in U937 cells may be recommended for identification of channels and transporters responsible for alteration of cell ion and water balance in various situations.

## Methods

### Reagents

RPMI 1640 medium and fetal bovine serum (FBS, HyClone Standard) were purchased from Biolot (Russia). STS and ouabain were from Sigma-Aldrich (Germany), Percoll was purchased from Pharmacia (Sweden). The isotope ^36^Cl^−^ was from “Isotope” (Russia). Salts were of analytical grade and were from Reachem (Russia).

### Cell Cultures

U937 human histiocytic lymphoma cells were obtained from the Russian Cell Culture Collection (Institute of Cytology, Russian Academy of Sciences, cat. number 160B2). The cells were cultured in RPMI 1640 medium supplemented with 10% FBS at 37°C and 5% CO_2_ overlay. For the induction of apoptosis, the cells, at a density of 1 × 10^6^ cells per ml, were exposed to STS for 0.5–4 h. All the incubations were done at 37°C.

### Determination of Cell Ion and Water Contents

The experimental methods used in this work have been described in detail earlier (Yurinskaya et al., 2005a,b, 2011; Vereninov et al., 2007, 2008). In summary, the cells were pelleted in RPMI medium, washed five times with MgCl_2_ solution (96 mM) and treated with 5% trichloroacetic acid (TCA). TCA extracts were analyzed for ion content. Intracellular K^+^, Na^+^, and Rb^+^ contents were determined by flame emission on a Perkin-Elmer AA 306 spectrophotometer. To determine the intracellular Cl^−^, the cells were cultured for 90 min or more at 37°C in RPMI medium containing ^36^Cl^−^ (0.12 μCi ml^−1^). The radioactivity of ^36^Cl^−^ in TCA extracts was measured using a liquid scintillation counter (Beckman LS 6500). The intracellular Cl^−^ content was calculated, taking into account the specific activity of ^36^Cl^−^ (~2 counts min^−1^ μmol^−1^). The TCA precipitates were dissolved in 0.1 N NaOH and analyzed for protein by the Lowry procedure, with serum bovine albumin as a standard. The cell ion content was calculated in micromoles per gram of protein.

Cell water content was determined by measurements of the buoyant density of the cells in continuous Percoll gradient. Percoll solution was prepared according to the manufacturer's instructions, and a thick cell suspension (0.1–0.2 ml, ~3 × 10^6^ cells) was placed on the solution surface and centrifuged for 10 min at 400 × g (MPW-340 centrifuge, Poland). The buoyant density of the cells was estimated using density marker beads (Sigma-Aldrich, Germany). The water content per gram of protein, *v*_prot_, was calculated as *v*_prot._ = (1-ρ*/*ρ_dry_)/[0.72(ρ-1)], where ρ is the measured buoyant density of the cells and ρ_dry_ is the cell dry mass density, which was 1.38 g ml^−1^. The proportion of protein in dry mass was 72%.

Cellular ion concentration was calculated from the values of ion and water content per gram of protein. In view that intracellular water content in living cells is much more variable than ion content, and because the ions and protein were assayed in one sample whereas cell water content in parallel samples the ion content per gram of protein should be considered as more reliable than ion concentration in cellular water.

### The Na^+^/K^+^ Pump Rate Coefficient Determination

The pump rate coefficient was determined based on the assay of the ouabain-sensitive Rb^+^ influx and cell Na^+^ content. The cells were incubated in medium with 2.5 mM RbCl and with or without 0.1 mM ouabain for 10 min. The rate coefficient of the Na^+^/K^+^ pump (*beta*) was calculated as the ratio of the Na^+^ pump efflux to the cell Na^+^ content given the assumption of the simple linear dependence of Na^+^ efflux on cell Na^+^ in the studied range of concentrations. The pump Na^+^ efflux was calculated from ouabain-sensitive (OS) Rb^+^ influx assuming proportions of [Rb]_o_ and [K]_o_ of 2.5 and 5.8 mM, respectively, and Na/K pump flux stoichiometry of 3:2.

### Calculation of the Monovalent Ion Flux Balance

The mathematical model of cell ion homeostasis and the algorithm of the numerical solution of the flux balance were described in detail earlier (Vereninov et al., 2014, 2016). The reader can reproduce all presented computed data and perform new calculations for various parameters by using the executable file to programme code BEZ01B (How to use programme code BEZ01B.zip in Supplementary Material). This software differs from the previous BEZ01 by the additional parameter *kb*, which characterizes a decrease in the pump rate coefficient β with time. Symbols and definitions used are shown in Table 1. The input data used in calculation as file DATAB.txt in Supplementary Material (see BEZ01B.zip in Supplementary Material) are the following: extracellular and intracellular concentrations (*na0, k0, cl0*, and *B0*; *na, k*, and *cl*); *kv*; the pump rate coefficient (β); the pump Na/K stoichiometric coefficient (γ); parameter *kb*; channel permeability coefficients (*pna, pk, pcl*); and the rate coefficients for the Na^+^-Cl^−^ (NC), K^+^-Cl^−^ (KC), and Na^+^-K^+^-2Cl^−^ (NKCC) cotransporters (*inc, ikc, inkcc*). The results of our computations appear in the file RESB.txt in Supplementary Material (Table 2) after running the executable file.

**Table 1:** Symbols and definitions.

**Definitions**	**In text and figures**	**In files DATAB.txt, RESB.txt**	**Units**
Ion species	Na^+^, K^+^, Cl^−^, Rb^+^	Na, K, Cl	
Types of cotransport	NC, NKCC, KC		
Concentration of ions in cell water or external medium	[Na]_i_, [K]_i_, [Cl]_i_, [Na]_o_, [K]_o_, [Cl]_o_	*na, k, cl, na0, k0, cl0*	mM
External concentrations of membrane-impermeant non-electrolytes such as mannitol introduced in artificial media	*B0*		mM
Intracellular ion contents	*Na_*i*_, K_*i*_, Cl_*i*_*		mmol, may be related to g cell protein or cell number, etc.
Intracellular content of membrane-impermeant osmolytes	*A*		mmol, may be related to g cell protein or cell number, etc.
Cell water volume	*V*		ml, may be related to g cell protein or cell number, etc.
Membrane-impermeant osmolyte concentration in cell water	A/V*1,000		mM
Cell water content per unit of *A*	*V/A*		ml mmol^−1^
Mean valence of membrane-impermeant osmolytes, *A*	*z*	*z*	Dimensionless
Permeability coefficients	pNa, pK, pCl	*pna, pk, pcl*	min^−1^
Pump rate coefficient	β	*beta*	min^−1^
Na/K pump flux stoichiometry	γ	*gamma*	Dimensionless
Membrane potential, MP	*U*		mV
Dimensionless membrane potential *u* = *U*F/RT	*u*		Dimensionless
Net fluxes mediated by cotransport	*J*_NC_*, J*_NKCC_*, J*_KC_	*NC, KC, NKCC*	μmol min^−1^ (ml cell water)^−1^
Na efflux via the pump	–β[Na]_i_	*PUMP*	μmol min^−1^ (ml cell water)^−1^
K influx via the pump	β[Na]_i_/γ	*PUMP*	μmol min^−1^ (ml cell water)^−1^
Net fluxes mediated by channels		*Channel*	μmol min^−1^ (ml cell water)^−1^
Unidirectional influxes of Na, K or Cl via channels or cotransport		*IChannel, INC, IKC, INKCC*	μmol min^−1^ (ml cell water)^−1^
Unidirectional effluxes of Na, K, or Cl via channels, or cotransport		*EChannel, ENC, EKC, ENKCC*,	μmol min^−1^ (ml cell water)^−1^
Time derivatives of concentrations		*prna, prk, prcl*	mM min^−1^
Cotransport rate coefficients	*i_*NC*_, i_*KC*_*	*inc, ikc*	ml μmol^−1^ min^−1^
	*i_*NKCC*_*	*inkcc*	ml^3^ μmol^−3^ min^−1^
Ratio of “new” to “old” media osmolarity when the external osmolarity is changed		*kv*	Dimensionless
Number of time points between output of results		*hp*	Dimensionless
Transmembrane electrochemical potential difference for Na^+^, K^+^, or Cl^−^	Δμ_Na_, Δμ_K_, Δμ_Cl_	*mun, muk, mucl*	mV
Ratio of ouabain-sensitive to ouabain-resistant Rb^+^ (K^+^) influx	OSOR	*OSOR*	Dimensionless
Parameter β decreases linearly with time with coefficient	*kb*		min^−1^

Table 2Results of computation.

**Table:** 

***t***	***U***	***na***	***k***	***cl***	***V/A***	***mun***	***muk***	***mucl***	***prna***	***prk***	***prcl***
**(A) TIME COURSE OF VARIABLES**											
0	−44.3	33.0	152.0	45.0	12.50	−82.8	42.9	19.0	0.00000	0.00000	0.00000
24	−44.5	36.1	148.9	45.2	12.52	−80.7	42.2	19.3	0.07612	−0.07696	0.00308
^**^											
216	−44.7	38.0	147.0	45.1	12.51	−79.5	41.7	19.4	0.00007	0.00004	−0.00040
240	−44.7	38.0	147.0	45.1	12.51	−79.5	41.7	19.4	0.00004	0.00005	−0.00032

**Table:** 

**(B) PARAMETER VALUES (COPY OF THE FILE DATAB.TXT)**											
***na0***	***k0***	***cl0***	***B0***	***kv***	***na***	***k***	***cl***	***beta***	***gamma***
140	5.8	116	48.2	1.0	33	152	45	0.039	1.5
**pna**	**pk**	**pcl**	**inc**	**ikc**	**inkcc**	**hp**	**kb**		
0.00382	0.022	0.0091	3E-5	0	0	240	0

**Table:** 

**(C) FLUX BALANCE UNDER THE BALANCED STATE**					
**Net flux**	**PUMP**	**Channel**	**NC**	**KC**	**NKCC**
Na	−1.4811	1.0451	0.4359	0.0000	0.0000
K	0.9874	−0.9878	0.0000	0.0000	0.0000
Cl	0.0000	−0.4363	0.4359	0.0000	0.0000
**Influx**	**PUMP**	**IChannel**	**INC**	**IKC**	**INKCC**
Na	0.0000	1.1012	0.4872	0.0000	0.0000
K	0.9874	0.2627	0.0000	0.0000	0.0000
Cl	0.0000	0.4082	0.4872	0.0000	0.0000
**Efflux**	**PUMP**	**EChannel**	**ENC**	**EKC**	**ENKCC**
Na	−1.4811	−0.0561	−0.0513	0.0000	0.0000
K	0.0000	−1.2505	0.0000	0.0000	0.0000
Cl	0.0000	−0.8445	−0.0513	0.0000	0.0000
***z***	**OSOR**	A/V*1000			
−1.75	3.76	79.95			

*Transition of the system to the balanced state as displayed in the file RESB.txt in Supplementary Material. The values similar to those for U937 cells with a rather high U and Δμ_Cl_ were chosen for this example of a transition to the balanced state. The displayed values of fluxes as well as OSOR correspond to the latest time point. The values of fluxes for other moments can be obtained by setting the necessary time interval with the hp value. The presented flux data do not include the fluxes involved in one-for-one exchange because they have no effect on cell ion or water content or MP and can be ignored here. The flux data clearly demonstrate how the net fluxes via different channels and transporters compensate for each other and come finally, under appropriate conditions, to a fully balanced ion distribution when the balance of influx and efflux is achieved for all ion species and prna, prk, prcl tend to zero*.

** *Time points not shown*.

The flux equations were:

$$\begin{array}{l} \begin{array}{l} \frac{dNa_{i}}{dt}=V\{(p_{Na}u({\left[Na\right]}_{i}\exp(u)-{\left[Na\right]}_{o})/g-\text{β}{\left[Na\right]}_{i}\\ \;+J_{NC}+J_{NKCC}\}\\ \frac{dK_{i}}{dt}=V\{(p_{K}u({\left[K\right]}_{i}\exp(u)-{\left[K\right]}_{o})/g+\text{β}{\left[Na\right]}_{i}/\gamma \\ \;+J_{NKCC}+J_{KC}\}\\ \frac{dCl_{i}}{dt}=V\{(p_{Cl}u({\left[Cl\right]}_{o}\exp(u)-{\left[Cl\right]}_{i})/g+J_{NC}\\ \;+J_{KC}+2J_{NKCC}\} \end{array} \end{array}$$

Here *u* is the dimensionless membrane potential (MP) related to absolute values *U* (mV) as *U* = *u*RT/F = 26.7*u* for 37°C and *g* = 1 − exp(*u*). The left-hand sides of these three equations represent the rates of change in the cell ion content, *Na*_*i*_ = [Na]_i_*V, K*_*i*_ = [K]_i_*V, Cl*_*i*_ = [Cl]_i_*V*. The right-hand sides express fluxes via channels, the Na/K pump, and cotransporters. The rate coefficients *p*_Na_, *p*_K_, *p*_Cl_ characterizing channel ion transfer are similar to the Goldman's coefficients. Fluxes *J*_NC_*, J*_KC_*, J*_NKCC_ depend on internal and external ion concentrations as

$$\begin{array}{l} J_{NC}=i_{NC}\left({\left[Na\right]}_{o}{\left[Cl\right]}_{o}-{\left[Na\right]}_{i}{\left[Cl\right]}_{i}\right)\\ J_{KC}=i_{KC}\left({\left[K\right]}_{o}{\left[Cl\right]}_{o}-{\left[K\right]}_{i}{\left[Cl\right]}_{i}\right)\\ J_{NKCC}=i_{NKCC}\left({\left[Na\right]}_{o}{\left[K\right]}_{o}{\left[Cl\right]}_{o}{\left[Cl\right]}_{o}-{\left[Na\right]}_{i}{\left[K\right]}_{i}{\left[Cl\right]}_{i}{\left[Cl\right]}_{i}\right) \end{array}$$

Here *i*_*NC*_*, i*_*KC*_, and *i*_*NKCC*_ are the rate coefficients for cotransporters (*inc, ikc, inkcc* in program symbols, Vereninov et al., 2014). Transmembrane electrochemical potential differences for Na^+^, K^+^, and Cl^−^ were calculated as: Δμ_Na_ = 26.7·ln([Na]_i_ /[Na]_o_)+*U*, Δμ_K_ = 26.7·ln([K]_i_ */*[K]_o_)+*U*, and Δμ_Cl_ = 26.7·ln([Cl]_i_ */*[Cl]_o_)-*U*, respectively. The values of electrochemical potential differences for Na^+^, K^+^ and Cl^−^, denoted in program symbols as *mun, muk*, and *mucl*, are important because they show the driving force and the direction of ion movement via channels and transporters under the indicated conditions. It is the changes in Δμ_Na_, Δμ_K_, and Δμ_Cl_ that are responsible for the possible fast effects of MP on ion fluxes via “electroneutral” transporters.

### Statistical Analysis

Experimental data are presented as the mean ± SEM. *P* < 0.05 (Student's *t* test) was considered statistically significant. Reliability of the calculated data is discussed further.

## Results

### Computational Approach to the Solution of the Problem of How the Entire Cell Ion and Water Balance Depends on the State of Various Channels and Transporters

The first of the two main aims of the present study is the demonstration of the computational approach to the solution of the problem of how the entire cell ion and water balance depends on the parameters of various channels and transporters. The second aim is the analysis of the ion and water balance changes during apoptosis in real U937 cells. This aim is an example of using the developed approach. Some background points should be considered first. The basic mathematical model used in our approach is similar to the known model developed by pioneers for analysis of ion homeostasis in normal cells (Jakobsson, 1980; Lew and Bookchin, 1986; Lew et al., 1991). Our algorithm of the numerical solution of the flux equations and basic software was published earlier (Vereninov et al., 2014, 2016). Some minor differences in mathematical models used by previous authors consist in the number of transporters included in the calculations. Only the Na^+^/K^+^ pump and electroconductive channels were considered in the early computational studies of cell ion balance. Lew and colleagues were the first who found that the Na^+^/K^+^ pump and electroconductive channels cannot explain monovalent ion flux balance in human reticulocytes because they cannot explain the non-equilibrial Cl^−^ distribution under the balanced state without NC (Lew et al., 1991). Cotransporters NC and KC were investigated by Hernández and Cristina (1998). The NKCC cotransport was included in ion balance modeling in cardiomyocytes (Terashima et al., 2006). Our software accounts for Na^+^, K^+^, and Cl^−^ channels, the Na^+^/K^+^ pump and the NC, KC and NKCC cotransporters. We found that NC is necessary as a rule in the calculation of the resting monovalent ion flux balance in U937 cells, while NKCC and KC are not. Nevertheless, the parameters characterizing these two transporters are present in our code, and fluxes via transporters can be accounted for if these parameters differ from zero.

Two points may worry experimentalists. First, the Na^+^/K^+^ pump activity is characterized by a single rate coefficient. However, a set of ion binding sites are known in the pump, and its operation kinetics in biochemical studies is described commonly by more than one parameter. The single rate coefficient is used because of the evaluation of the properties of all the ion binding sites of the pump in experiments in whole cells is infeasible and because it appears to be quite sufficient for the calculation of entire-cell ion homeostasis. This idea was demonstrated by the quantitative prediction of the dynamics of monovalent ion redistribution after stopping the Na^+^/K^+^ pump (Vereninov et al., 2014, 2016). Single rate coefficients for characterizing the ion carriage kinetics via transporters are commonly used for the same reason. The second point causing disapproval might be that an integral permeability coefficient is used in the calculation of the flux balance for all Na^+^ or K^+^ or Cl^−^ channels, whereas a great variety of channels for each ion species is located in the plasma membrane. The single permeability coefficients are commonly used in the analysis of the entire-cell flux balance because in an analysis of such a complex system with many channels and transporters, the matter of primary importance is to understand whether ion flux changes due to alteration of the force driving the ions or by properties of the channels or transporters *per se*.

### Computation of Ion Flux Balance in Cells Similar to U937 Cells

Parameters in absolute units are used in our calculations. Their initial, “standard,” values are obtained from the calculation based on the distribution of monovalent ions and ouabain-sensitive Rb^+^(K^+^) influx measured in cells under normal physiological conditions and the cell balanced state. The parameters varied until the tested values give a calculated entire ion and water homeostasis similar to that in real cells. The “standard” parameters can vary in real cells depending on the cell physiological state, the age of the culture, the conditions of cell cultivation, etc.

**Figure 1:**
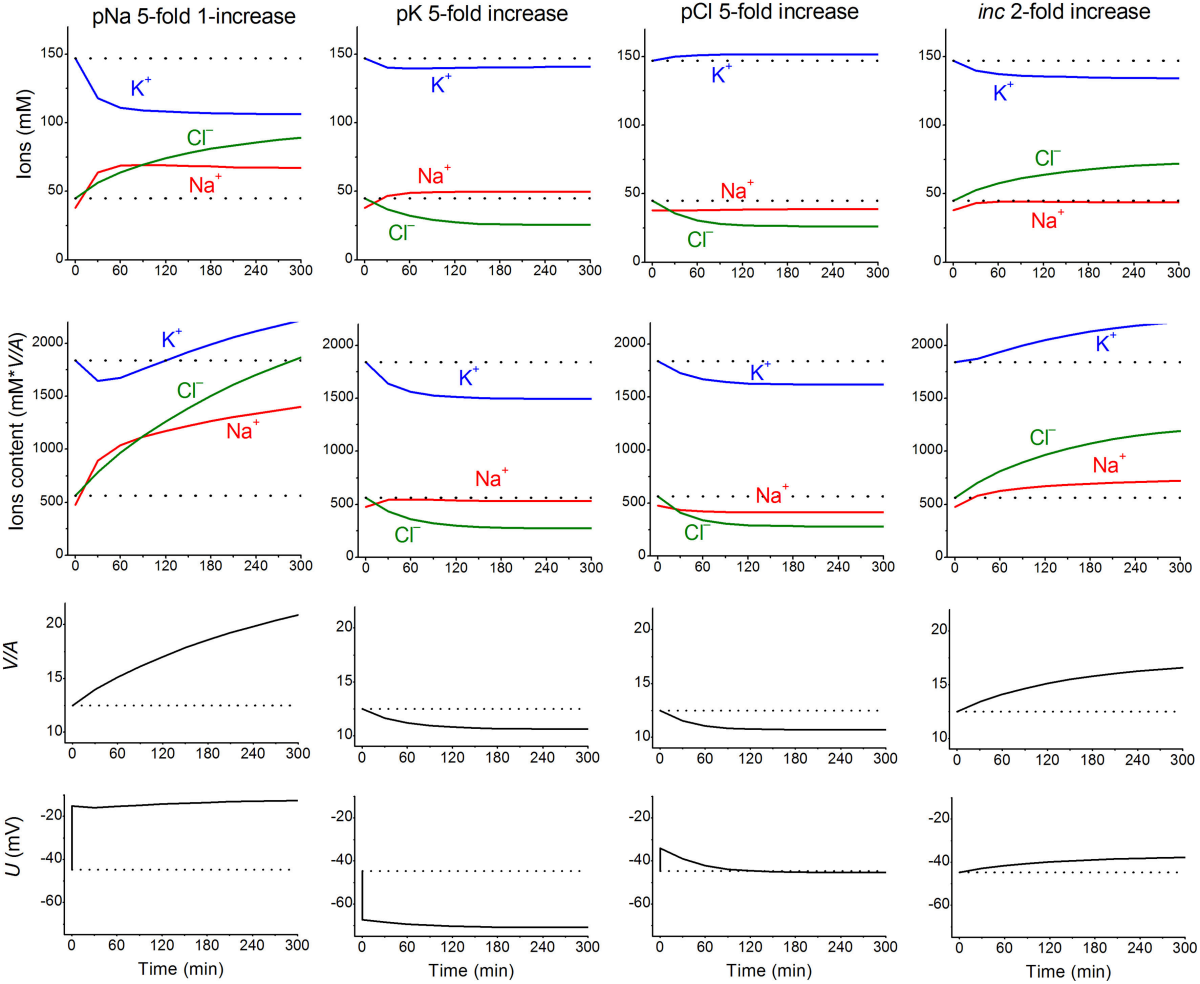
The calculated effects of an abrupt increase in the permeability coefficients of K^+^, Na^+^, Cl^−^ channels, or the NC cotransport rate coefficient on cell K^+^, Na^+^, and Cl^−^ content and concentrations, water-volume (*V/A*) and MP (*U*). The data were calculated by using the software BEZ01B. The initial parameters were *na0* 140, *k0* 5.8, *cl0* 116, *B0* 48.2, *kv* 1, *beta* 0.039, *gamma* 1.5, *pna*, 0.00382, *pk* 0.022, *pcl* 0.0091, *inc* 0.00003, *ikc* = *inkcc* = 0, *kb* 0, and *hp* 300, i.e., much like U937 cells; the changed parameters are shown on the plots.

Nevertheless, these parameters can remain invariant under a varying environment. We found that the kinetics of the disturbance of cell ion and water balance caused by blocking the Na^+/^K^+^ pump when the intracellular K^+^/Na^+^ ratio is highly changed and even reversed can be predicted sufficiently well by calculation with the invariant parameters (Vereninov et al., 2014, 2016). A set of examples is presented in Figure 1 to show how changes in a single channel or transporter species (one permeability coefficient or rate constant) can alter the intracellular concentrations of all major ions, cell water content and the MP. Unlike pNa and *i*_*NC*_, changes in pK or pCl lead, over the course of 60–100 min, to a new balanced state. Even this small set of examples demonstrates that some effects seem to be unexpected at first sight. Intracellular K^+^ concentration decreases monotonically with the pNa increase, while the intracellular K^+^ content decreases initially and increases further due to the superposition of the initial drop MP and the slow increase in cell water-volume. An increase in the coupled equivalent transport Na^+^ and Cl^−^ (*i*_*NC*_,) causes a decrease in cell K^+^ concentration, [K^+^], and, in contrast, an increase in cell K^+^ content because of changes in cell water volume and in MP. The [K^+^] and MP are shifted in this case in opposite directions. It should be stressed that the effects of parameter variation are highly dependent on the cell species. Our previous paper presented the typical dependences for the cells with high MP and high intracellular K/Na ratio, for the cells with low MP and high K/Na ratio (high potassium erythrocytes) and for the low-MP and low-K/Na-ratio cells (low-potassium erythrocytes of some carnivores and ruminants) (Vereninov et al., 2014). Cells such as U937 and their variant with relatively high Δμ_Cl_ (19.4 mV) are chosen as an example in Figure 1 to make the pCl effect more obvious.

**Figure 2:**
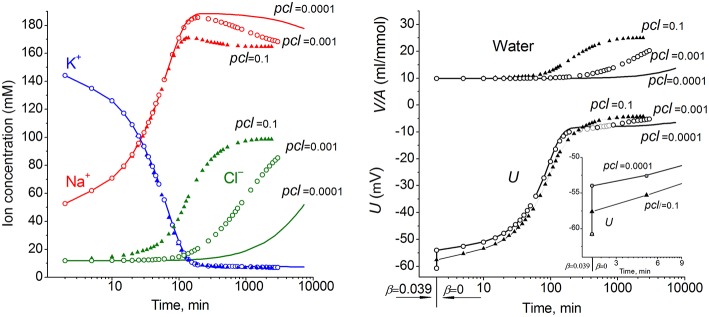
The effect of pCl on the time course of the ion and water balance disturbance caused by turning off the pump. The data were calculated by using the software BEZ01B with the following parameters: *na0* 140, *k0* 5.8, *cl0* 116, *B0* 48.2, *kv* 1, *na* 52.6, *k* 144.2, *cl* 11.9, *beta* 0 (at the initial balanced value of 0.039), *gamma* 1.5, *pna* 0.006, *pk* 0.06, *pcl* 0.1 (triangles) or 0.001 (circles) or 0.0001 (solid lines), *inc* = *ikc* = *inkcc* = 0, *kb* 0.

The ion and water redistribution caused in U937 cells by stopping the Na^+^/K^+^ pump was studied earlier *in silico* and in an experiment (Vereninov et al., 2014, 2016). Here, it is interesting to demonstrate this case as an example of asynchrony in changes of K^+^, Na^+^, and Cl^−^ after blocking the pump (Figure 2). In the earlier stage, the electroneutrality of the net ion fluxes is achieved mainly by the balance of fluxes K^+^ outward and Na^+^ inward via channels, whereas, later, the Cl^−^ influx becomes significant. There is no alteration of total intracellular osmolytes during the equal K^+^/Na^+^ exchange, and it is for this reason that no swelling occurs after blocking the pump for a rather long time. It should be stressed that no specific carrier is responsible for the balanced K^+^/Na^+^ exchange. This result is realized via electroconductive channels only due to the dependence of fluxes on MP. The long-term balanced state in monovalent ions and water distribution after stopping the pump is unattainable, and cell swelling will go on infinitely. However, the kinetics of the entire process may be different in dependence on the pCl level. It should be noted that cell water content and intracellular concentration are changing synchronously. It is the low Cl^−^ channel permeability that saves real cells from swelling for a long time after blocking the Na^+^/K^+^ pump.

**Figure 3:**
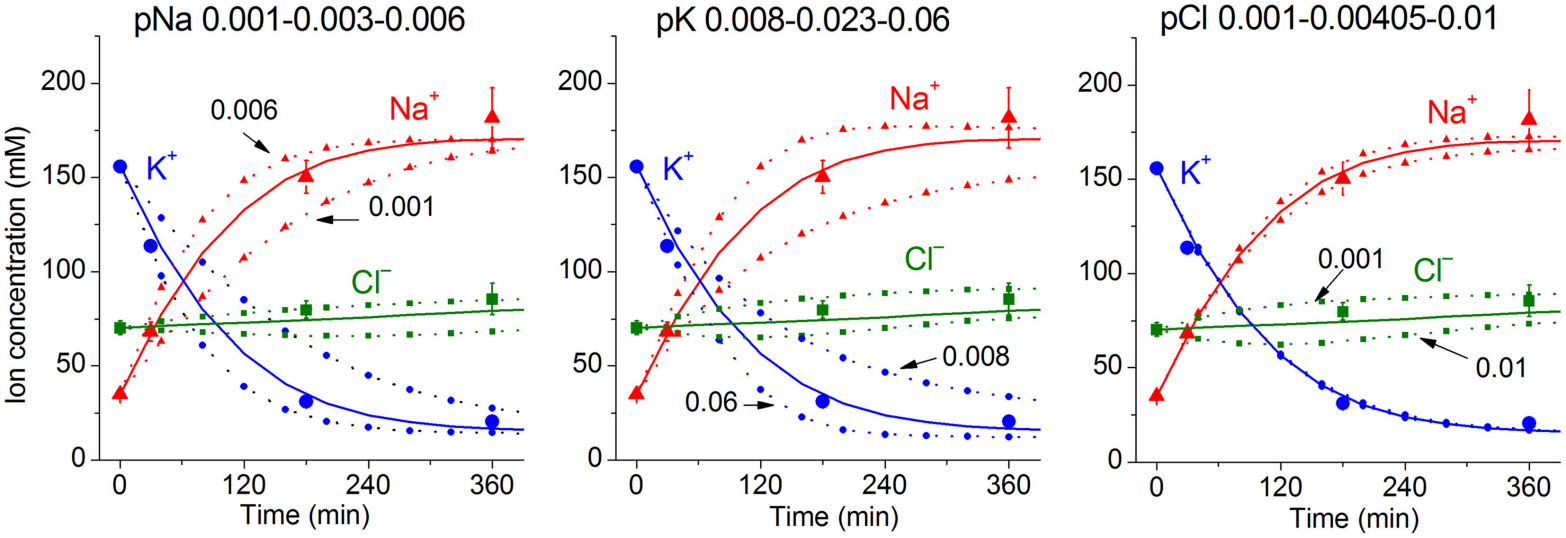
Dependence of calculated K^+^, Na^+^, Cl^−^ redistribution dynamics after blocking the pump on K^+^, Na^+^, and Cl^−^ channel permeabilities. Experimental data are shown by the large symbols. Solid lines—data calculated for the following parameters: *na0* 140, *k0* 5.8, *cl0* 116, *B0* 48.2, *kv* 1, *na* 35, *k* 156, *cl* 70, *beta* 0.001, *pna* 0.00301, *pk* 0.023, *pcl* 0.00405, *inc* 3.4E-5, *ikc* = *inkcc* = 0, (before blocking the pump *beta* was 0.039), *gamma* 1.5. Dotted lines with small symbols—data calculated for parameter values indicated above the graphs.

Due to a large number of adjustable parameters and experimental observations, the usual statistical verification of reliability of calculated data is not applicable. In order to determine the sensitivity of results to experimental errors and the choice of parameters, one can simply repeat calculations for slightly different input values. It should be noted that sensitivity of results to such changes must be determined for specific conditions. Figure 3 shows the effects of changes in the permeability of Na^+^, K^+^, and Cl^−^ channels on the time course of ion redistribution caused by blocking of the Na^+^/K^+^ pump.

### Changes in K^+^, Na^+^, Cl^−^, and Water Contents During Early Apoptosis in U937 Cells Induced by STS

Most data on the redistribution of monovalent ions during apoptosis relates to the 4–5 h stage (see references in Arrebola et al., 2005a). Our simultaneous determination of K^+^, Na^+^, Cl^−^, and water contents in U937 cells treated with STS for 4 h was published earlier (Yurinskaya et al., 2011). The data related to this apoptosis stage confirmed the osmotic mechanism of AVD, i.e., they showed that the water loss was caused mostly by the loss of the total monovalent ion content and much less by a decrease in content of the “impermeant intracellular anions,” *A*^−^. The initial changes in all major monovalent ions and water content during apoptosis have been studied much less, although the early cell shrinkage is supposed to be crucial for triggering apoptosis. Our current data on the changes in ion and water content during STS apoptosis in U937 cells with the earliest time point 30 min are presented in Table 3. The values of the independently determined ion content and water content correspond to the osmotic mechanism of AVD at the early stages as well as at the 4 h stage studied before. The data on water content in Table 3 were obtained by the best method, i.e., by cell buoyant density. These data agree well with the data obtained by using a Coulter counter and flow cytometer (Yurinskaya et al., 2017). Calculation of the changes in K^+^, Na^+^, and Cl^−^ net fluxes underlying the changes in cell ion and water content shows that for the first hour, the K^+^ loss is electrically balanced predominantly by the Cl^−^ loss, whereas later it is mostly balanced by the Na^+^ gain (Table 4, last columns).

**Table 3:** Changes in K^+^, Na^+^, Cl^−^, and water contents during the early stages of STS apoptosis in U937 cells.

**Time**	**K^**+**^**	**Na^**+**^**	**Cl^**−**^**	***A*^**−**^**	**Water**
**min**	**μmol** **·(g prot.)**^****−1****^				**ml/g**
0	712 ± 22	192 ± 8	246 ± 11	658	6.08 ± 0.08
30	615 ± 12	175 ± 10	133 ± 4	657	5.37 ± 0.21
120	595 ± 13	179 ± 4	109 ± 5	665	4.70 ± 0.05
240	493 ± 21	261 ± 5	117 ± 4	637	4.85 ± 0.08

*Means ± SEM from three independent experiments with duplicate determinations*.

**Table 4:** Changes in cell [K^+^], [Na^+^], [Cl^−^], MP (U) and net fluxes calculated upon a linear decrease in the pump rate coefficient and stepwise changes in the K^+^, Na^+^, and Cl^−^ channel permeability corresponding to the experimental data on apoptotic alteration of K^+^, Na^+^, and Cl^−^ concentrations and pump fluxes.

**Time, min**	***beta***	***pk***	***pna***	***pcl***	***U***	***na***	***k***	***cl***	***V/A***	***mun***	***muk***	***mucl***	**Net fluxes**		
													**Na^**+**^**	**K^**+**^**	**Cl^**−**^**
Before	0.029	0.0115	0.0041	0.0125	−29.9	32	117	40	8.26	−69.3	50.3	1.5	0	0	0
0	0.029	0.03	0.003	0.068	−34.6	32.0	117	40.0	8.26	−74.0	45.6	6.2	0	0	0
10	0.028				−38.5	32.3	116.4	33.5	7.82	−77.7	41.6	5.3	−0.119	−0.622	−0.733
20	0.028				−42.0	32.7	115.7	28.4	7.51	−80.8	37.9	4.4	−0.065	−0.481	−0.540
30	0.027				−45.0	33.3	114.9	24.6	7.29	−83.3	34.8	3.5	−0.020	−0.366	−0.381
60	0.025	0.02			−44.8	33.8	114.3	22.3	7.16	−82.8	34.8	0.8	0.031	−0.079	−0.048
120	0.021				−45.1	37.7	110.3	21.7	7.13	−80.1	33.5	0.3	0.086	−0.078	0.008
^**^					……………………………………………….										
210	0.015				−43.2	47.2	100.9	23.2	7.21	−72.2	33.1	0.1	0.139	−0.110	0.029
240	0.013				−42.2	51.4	96.7	24.0	7.25	−68.9	33.0	0.1	0.163	−0.127	0.036

*The initial parameters were na0 140, k0 5.8, cl0 116, B0 48.2, kv 1, na 32, k 117, cl 40, gamma 1.5, inc 0.000003, and kb 0.000068. The changed parameters, including beta, pna, pk, and pcl, are shown in the Table*.

** *Time points not shown. The data were obtained by using the code BEZ01B. The time of channel alteration is indicated by horizontal lines. Outward net fluxes are defined as negative*.

The apoptotic changes in ion content obtained in our study by flame photometry and radiotracer assay are very close to the data obtained by the X-ray microanalysis in U937 cells during several types of apoptosis, including early STS apoptosis (Arrebola et al., 2005b, 2006). Unlike that X-ray microanalysis study, we could more easily validate changes in cell water content during apoptosis and therefore better estimate ion concentrations per cell water volume. This approach enabled us to calculate the entire cell electrochemical system and, in this way, to identify channels and transporters critical for AVD and underlying monovalent ion redistribution.

### Matching the Real and Calculated Changes in Cell K^+^, Na^+^, and Cl^−^ Concentrations During Apoptosis

The real changes in Na^+^, K^+^, Cl^−^ and water contents during STS apoptosis in U937 cells differ from the calculated example presented in Figure 1. Evidently, other changes in rate parameters during the transient process can occur in real cells. Indeed, a decrease in the Na^+^/K^+^ pump activity is a peculiar feature of apoptosis and has been revealed without any computation, particularly in U937 cells treated with STS (Arrebola et al., 2005a,b; Vereninov et al., 2008; Yurinskaya et al., 2010, 2011). The rate coefficient of the Na^+^/K^+^ pump can be calculated by OS Rb^+^ influx and intracellular Na^+^ content (Vereninov et al., 2014, 2016). We found that the OS Rb^+^ influx for the first 4 h of STS apoptosis in U937 cells decreased mostly linearly (Yurinskaya et al., 2010).

The linear decrease in the pump rate coefficient with time was accounted for in the current programme code BEZ01B. Figure 3A shows the transient process during STS apoptosis in U937 cells calculated under the assumption that the pump rate coefficient decreases linearly due to the decrease in the coefficient *kb*, as found by OS-Rb^+^ influx assay in experiment. The values calculated for this simplest model (lines) correspond approximately to the real ion concentrations (symbols) for K^+^ (circles) and Na^+^ (triangles) but differ significantly for Cl^−^ (squares). The additional assumption that triggering apoptosis is accompanied by stepwise increases in pCl and pK and by a slight decrease in pNa improves the agreement between calculated and real values for Cl^−^ in the first 30–40 min, but not later (Figure 3B). A change in pCl alone becomes ineffective because of the small Δμ_Cl_. The agreement may be obtained for the whole 4 h time interval by assuming that pK further decreases (Figure 3C). How unique is the found fitting? By trial and error, we found that a pNa decrease and a pK increase alone without a pCl increase could be sufficient to get agreement between real and calculated chloride concentrations for the first 30 min. However, this case should be rejected because the value OSOR becomes unacceptably low.

**Figure 4:**
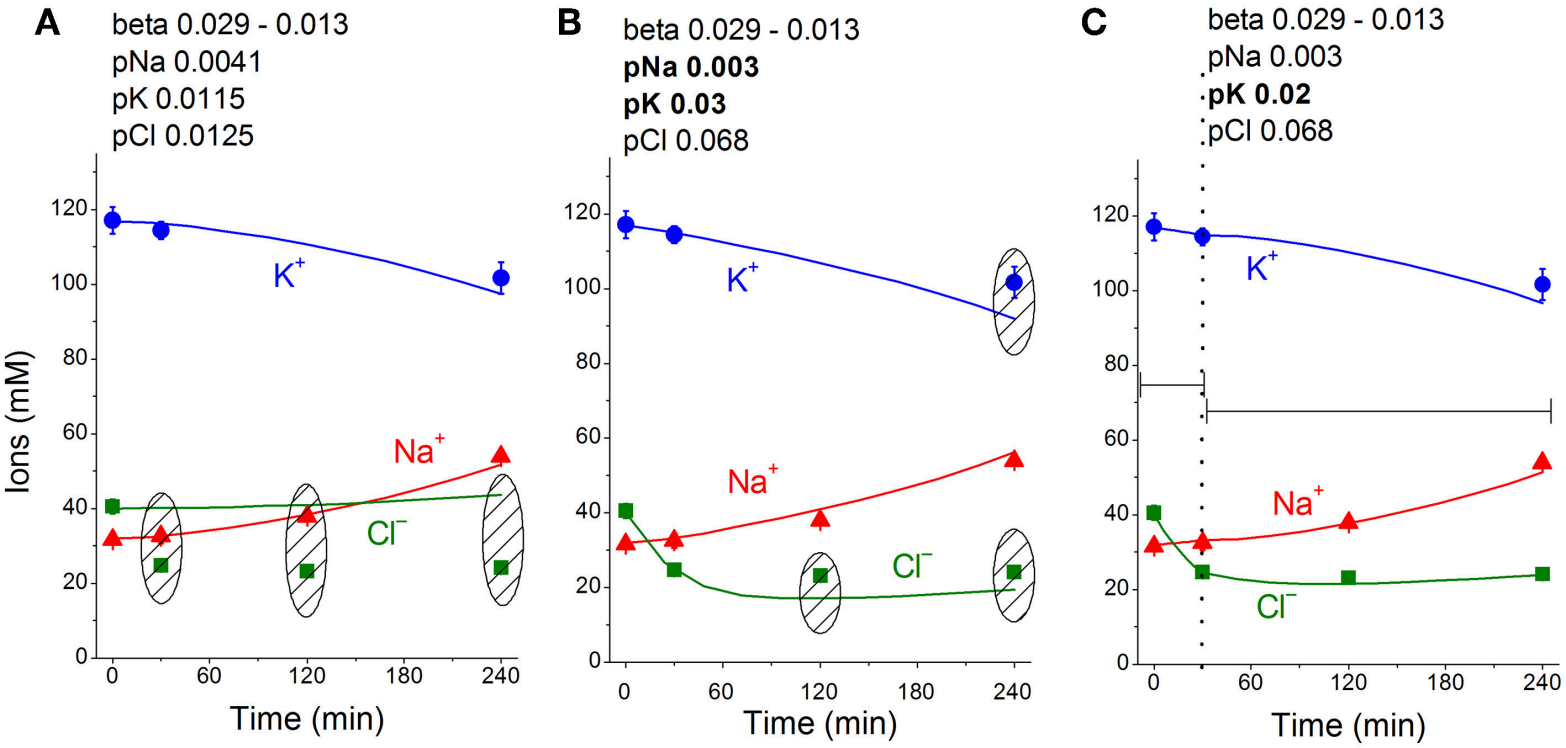
Time course of [K^+^], [Na^+^] and [Cl^−^] in real U937 cells treated with 1 μM STS (symbols) and calculated (lines) for different parameter datasets. Symbols—experimental data, means ± SEM from three independent experiments with duplicate determinations. Small SEM values are masked by symbols. Lines—calculated data obtained for the parameters indicated on the graphs. The initial parameters were *na0* 140, *k0* 5.8, *cl0* 116, *B0* 48.2, *kv* 1, *na* 32, *k* 117, *cl* 40, *beta* 0.029, *gamma* 1.5, *pna* 0.0041, *pk* 0.0115, *pcl* 0.0125, *inc* 0.000003, *ikc* = *inkcc* = 0, and *kb* 0.000068. The changed parameters are shown in the layers head. **(A)** Linear decrease in *beta* only. **(B)** Decrease in *beta* and changes in *pna, pk*, and *pcl*. **(C)** Additional decrease in *pk*. Shaded regions show significant disagreement of experimental and predicted values. The calculated data were obtained by using code BEZ01B.

The joint effect of pK, pNa and pCl shift is interesting. The cells shown in Figure 4 had initially a rather low *U* (−29.9 mV) and a *mucl* of ~1.5 mV under the normal state (Table 4). The variation of pCl alone at so small a *mucl* has no significant effect. A decrease in pNa hyperpolarizes cells promptly, and an increase in pK alone hyperpolarizes cells as well (Table 5). As a result, the pCl increase becomes effective and sufficient to get both the necessary agreement between real and calculated chloride concentrations for the initial 30–40 min and the necessary OSOR.

**Table 5:** The effects of pK, pNa, and pCl shifts at the initial stage of apoptosis on *U, mucl*, and ion concentrations.

***t***	***pk***	***pna***	***pcl***	***U***	***na***	***k***	***cl***	***V/A***	***mun***	***muk***	***mucl***
*pk* shift											
Initial	0.0115	0.0041	0.0125	−29.9	32.0	117.0	40.0	8.26	−69.3	50.3	1.5
0	**0.03**	0.0041	0.0125	**−41.1**	32.0	117.0	40.0	8.26	−80.6	39.1	**12.7**
15				−42.1	35.1	113.7	36.6	8.03	−79.0	37.4	11.3
30				−42.9	37.2	111.5	33.8	7.83	−78.3	36.0	10.0
45				−43.6	38.6	110.0	31.5	7.69	−78.0	34.9	8.8
60				−44.2	39.2	109.0	29.5	7.57	−78.0	34.1	7.7
*pna* shift											
Initial	0.0115	0.0041	0.0125	−29.9	32.0	117.0	40.0	8.26	−69.3	50.3	1.5
0	0.0115	**0.003**	0.0125	**−36.6**	32.0	117.0	40.0	8.26	−76.0	43.6	**8.2**
15				−37.2	30.6	118.3	38.0	8.12	−77.8	43.3	7.4
30				−37.8	29.7	119.1	36.2	8.00	−79.2	42.8	6.7
45				−38.4	29.2	119.5	34.6	7.90	−80.2	42.4	6.1
60				−38.9	28.9	119.7	33.3	7.81	−81.0	41.9	5.6
*pk* and *pna* shift											
Initial	0.0115	0.0041	0.0125	−29.9	32.0	117.0	40.0	8.26	−69.3	50.3	1.5
0	**0.03**	**0.003**	0.0125	**−44.8**	32.0	117.0	40.0	8.26	−84.2	35.4	**16.4**
15				−46.3	32.5	116.3	35.6	7.96	−85.3	33.8	14.7
30				−47.6	32.8	115.8	31.9	7.72	−86.4	32.3	13.1
45				−48.8	33.1	115.4	28.8	7.53	−87.3	31.1	11.5
60				−49.7	33.3	115.0	26.2	7.38	−88.1	30.0	10.0
*pk, pna* and *pcl* shift											
Initial	0.0115	0.0041	0.0125	−29.9	32.0	117.0	40.0	8.26	−69.3	50.3	1.5
0	**0.03**	**0.003**	**0.068**	−34.6	32.0	117.0	40.0	8.26	−74.0	45.6	**6.2**
15				−40.4	32.3	116.2	30.7	7.65	−79.6	39.7	4.9
30				−45.2	32.6	115.6	24.4	7.28	−84.2	34.7	3.6
45				−48.7	32.9	115.1	20.5	7.07	−87.4	31.1	2.5
60				−50.9	33.2	114.7	18.3	6.95	−89.4	28.8	1.6

*The initial parameters were as follows: na0 140, k0 5.8, cl0 116, B0 48.2, kv 1, beta 0.029, na 32, k 117, cl 40, gamma 1.5, inc 0.000003, kb 0*.

The *inc* and pCl parameters change cell water and [Cl]_i_ in opposite directions (Figure 1). However, we could not replace the pCl increase with the *inc* decrease in our fitting procedure, as the [Cl]_i_ decrease in the latter case is too small. We come to the conclusion that an increase in pCl is a critical factor for the complex water and ion rearrangement at the initial stage of STS apoptosis in U937 cells, whereas its role becomes less significant or even non-significant later.

We conclude that the redistribution of K^+^, Na^+^, and Cl^−^ underlying AVD in the studied U937 cells treated with STS is caused (1) by a progressive linear decrease in the pump rate coefficient from the initial 0.029 to 0.013 at 4 h, (2) by a significant increase in pCl (0.0125 to 0.068) and changes in pK (0.0115 to 0.03 and later 0.02), and (3) by a moderate decrease in pNa (0.0041 to 0.003). The most critical factors for changes in cell K^+^ and Na^+^ are suppression of the pump, an increase in pK and a decrease in pNa, whereas the early decreases in Cl^−^ and water content (early AVD) are associated primarily with an increase in pCl by about 5 times and an increase in pK by about 2.6 times.

**Figure 5:**
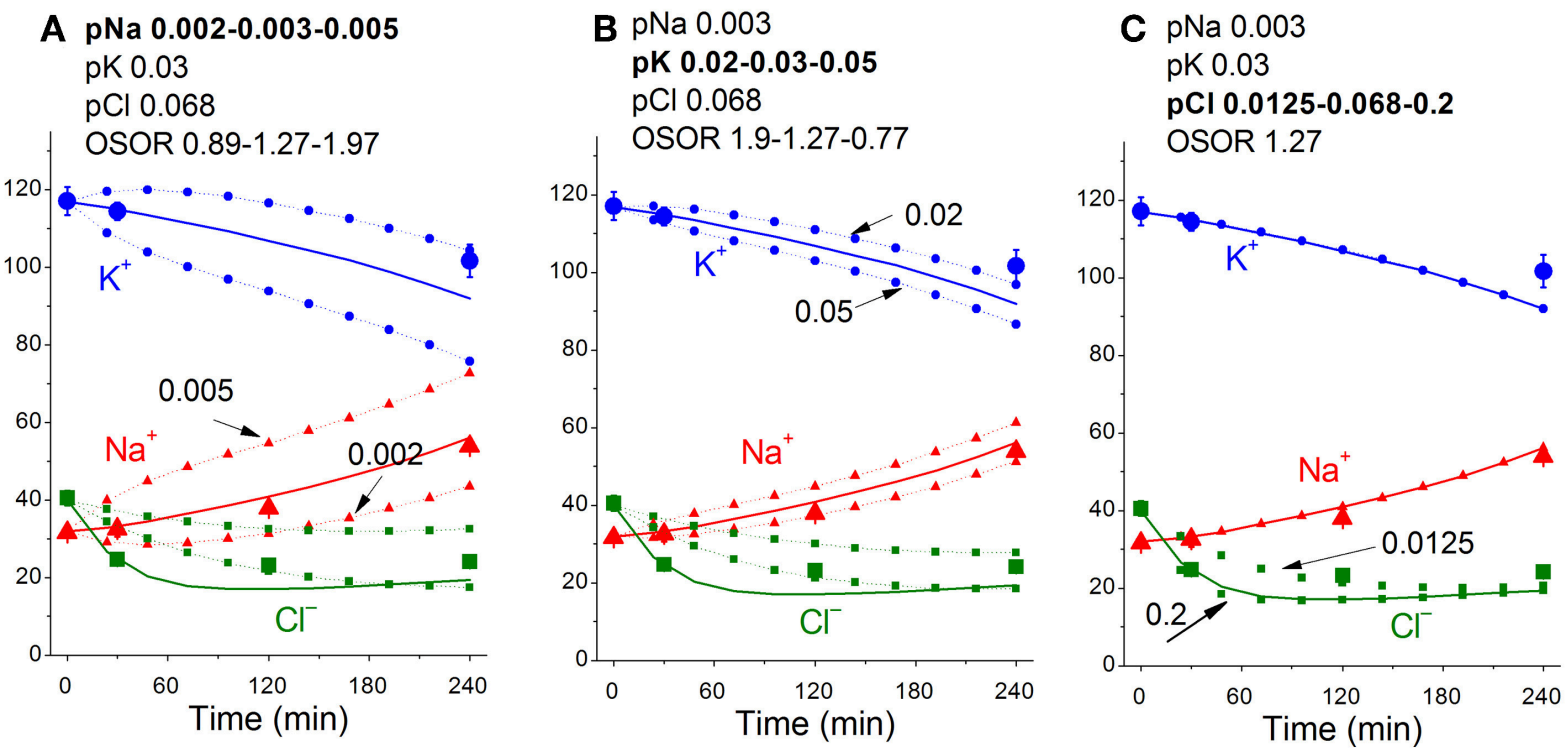
Dependence of the calculated K^+^, Na^+^, and Cl^−^ redistribution dynamics during STS-induced apoptosis on K^+^, Na^+^, and Cl^−^ channel permeabilities. Experimental data are shown by the large symbols. Solid lines—data calculated for the following parameters: *na0* 140, *k0* 5.8, *cl0* 116, *B0* 48.2, *kv* 1, *na* 32, *k* 117, *cl* 40, *beta* 0.029, *gamma* 1.5, *pna* 0.003, *pk* 0.03, *pcl* 0.068, *inc* 3E-6, *ikc* = *inkcc* = 0, *kb* 0.000068. Dotted lines with small symbols—data calculated for parameter values indicated above the graphs. OSOR values are given for a 4 h time point.

Figure 5 shows sensitivity of the calculated results to “trial” variations in the permeability coefficients of Na^+^, K^+^, and Cl^−^ channels in the case of apoptosis. Similar trials convinced us that the values of channel permeabilities and transporter parameters obtained by computation for apoptotic U937 cells are reliable and unique.

The calculated MP in the considered model of apoptosis was slightly hyperpolarizing (by 12 mV). Our preliminary results from flow cytometry using DiBAC_4_(3) did not show a significant change in MP under STS-induced apoptosis (unpublished data). These results differ from previous reports of MP depolarization during apoptosis, e.g., in the Fas-L induced apoptosis of Jurkat cells (Franco et al., 2006). Further studies are required to determine whether MP changes are highly dependent on the apoptosis inducer and/or on the cell species or whether cell depolarization occurs in more severe apoptosis.

## Discussion

Monovalent ion channels and transporters are involved in apoptosis (Burg et al., 2006; Lang et al., 2007; Hoffmann et al., 2009, 2015; Dezaki et al., 2012; Lang and Hoffmann, 2012; Orlov et al., 2013; Kondratskyi et al., 2015; Jentsch, 2016; Pedersen et al., 2016; Wanitchakool et al., 2016). However, this phenomenon may be caused by two reasons: because the monovalent ions are the major cell volume regulators and should be responsible for AVD only for this reason, or because they also play important roles in cell signaling by affecting MP. It is not easy to distinguish these two causes at present. We aimed to answer the question of how alteration of distinct channels and transporters affects the balance of monovalent ion fluxes across the cell membrane, cell water content and MP at apoptosis. We studied the time course of the monovalent ion balance redistribution during the first 4 h development of apoptosis induced in U937 cells treated with STS as the established model of apoptosis with significant AVD. Apoptosis in U937 cells is accompanied by rapid changes in light scattering and cell water (volume) balance, whereas the positive annexin test and intensive generation of apoptotic bodies are revealed, starting at 3–4 h (Yurinskaya et al., 2017). The identification of channels and transporters responsible for the observed changes in monovalent ion distribution, water balance and the pump fluxes was based on the computational modeling of these changes. Such an approach was applied here to study apoptosis for the first time, although the monovalent flux balance under the normal physiological state and during redistribution of ions due to stopping the pump has been calculated successfully before (see ref. in: Vereninov et al., 2014, 2016). Our previous code was modified currently to account for a continuous decrease in the pump rate coefficient.

One of the most detailed experimental studies of the kinetics of the monovalent ion balance rearrangement during apoptosis was performed by X-ray microanalysis and in U937 cells treated with STS in particular (Arrebola et al., 2005a,b). The experimental data obtained by flame emission and radiotracer assays in our study agree very well with the data obtained by this quite different method. Unfortunately, the accurate cell water content evaluation is hard to combine with the X-ray elemental microanalysis. Therefore, the complete mathematical model of the monovalent ion flux balance could not be developed using only those data.

Earlier we tried to relate the changes in ion and water contents to the monovalent fluxes in the Na^+^/*K*^+^ pump, K^+^, Na^+^, and Cl^−^ channels and certain cotransporters in U937 cells after 4 h of STS-induced apoptosis (Yurinskaya et al., 2011). We came to the conclusion that the Na^+^/*K*^+^ pump suppression accompanied by a decrease in Na^+^ channel permeability might be responsible for AVD under the considered conditions. However, our current computational tool had not been developed at that time, the experimental data were limited to single time point 4 h, and the assumption was used that the balanced monovalent ion distribution is reached at 4 h of STS-induced apoptosis in U937 cells. More complete current data show that the cells at the 4 h time point are far from the balanced state (Table 3). There is significant Na^+^ gain (0.163) that is by ¾ balanced by K^+^ leak (0.127) and ¼ by the gain of Cl^−^ (0.036).

Currently, we substantially revised and developed our previous conception of the participation of the major channels and transporters in AVD during the STS-caused apoptosis of U937 cells. It remains true that a slow decrease in the Na^+^/*K*^+^ pump activity is a primary factor responsible for AVD at the late (4 h) stage of apoptosis. Recalculation of the data published earlier (Yurinskaya et al., 2011) with use of the current programme code and without intricate hypotheses confirmed a decrease in pNa at 4 h. The current data show that the pNa decrease at 4 h is significant indeed. The most interesting and important phenomenon is a more than 5-fold increase in the Cl^−^ channel permeability, which is much more important at the early stage. It is remarkable that the effect of the pCl increase disappears further because of a decrease in intracellular Cl^−^ concentration and associated decrease in chloride electrochemical potential difference, Δμ_Cl_ (*mucl* in Table 4). The effects of the early increase in pK and a decrease in pNa on *U* are significant because they lead to an increase in Δμ_Cl_ that drives chloride outward. A large body of electrophysiological evidence published recently indicates that the state of the chloride channels can change upon initiation apoptosis (Hoffmann et al., 2015; Kondratskyi et al., 2015; Jentsch, 2016; Pedersen et al., 2016; Wanitchakool et al., 2016). However, there were no attempts to use these data for the quantitative description of early AVD.

The current computations show that changes in not a single type of channel but in K^+^, Na^+^, and Cl^−^ channels and in the Na^+^/K^+^ pump are responsible for the apoptotic ion balance alteration and that the effect of various channels and transporters on ion balance may be different at different stages of apoptosis. Certainly, the question arises how many parameters can provide accordance between the calculated and real data? The computation enables us to answer this question, although certain time may be needed. In the case of STS-induced apoptosis in U937 cells in our experiments, we can exclude alternative variants by taking into account additionally the value of OSOR, which appeared to be different in different parameter setups, giving sufficiently good accordance between the real and calculated data. In other cases, the problem could be solved probably not by using OSOR but by some other way. The computation shows also how the real behavior of cells should depend on the initial state of cells. Certainly, as soon as basic experimental data vary, the obtained numerical values of parameters will vary also.

A skeptical view is spread among the experimentalists on the using calculations in analysis of the ion flux balance in cells. There is also a great deal of sometimes convoluted discussion about the merits and validity of certain assumptions that need to be made for the models and real data to be reconciled. As believed it is very difficult to verify the models and their conclusions. In this regard, we should note the following. No hypothetical assumptions are used in our calculations as well as in similar studies of previous authors. The calculations are based on the “model-independent” theory. Two mandatory fundamental principles are used: macroscopic electroneutrality of ion transfer and osmotic balance between internal and external media in animal cells. All known types of monovalent ion pathways are accounted for in calculations of ion flux balance. These pathways are identified by the forces driving ions across the cell membrane which calculation is indisputable at present. The calculations do not depend on any peculiarity of molecular structure of proteins involved in ion transfer. Our study is not a modeling-imitation but a tool for solution the problem which cannot be solved currently without computation. The calculations include the interrelationships in the movement of ions across the membrane, due to their overall dependence on the membrane potential and contribution to the osmotic balance. This allows identifying changes in the pathways itself.

If a required set of experimental data is given a unique solution appears independently on any hypotheses on the number and types of channels and transporters which could present in the cell membrane. Our system of the flux equations accounts all currently known types of ion transfer across membrane characterized only by the ion driving forces: electrochemical potential difference for movement of single ion species (electrodiffusion through electroconductive channels), the sum of electrochemical potential differences for the linked movement of several species of ions (cotransport, countertransport), and a combination of the electrochemical and chemical potential differences in case of the Na^+^,K^+^-ATPase pump. Computer decides what number of transporting units of each type should be for implementation of two physically mandatory demands and which ionic pathways do not play a role under given conditions, particularly, at existent electrochemical gradients of each ion species. The mandatory demands are electroneutrality of the any macroscopic ion redistribution and osmotic balance between a distensible animal cell and the medium. Any hypothesis on the mechanism regulating cell water and ion content or membrane potential must be checked for these demands implementation. This cannot be done without computation in a system with a numerous species of ions and numerous ion pathways. Experimentalists avoid calculation and prefer using inhibitors and genetic cell modification simply because there is no sufficiently suitable tool for computation. We attempted to reduce computational tool deficiency.

As to general validation of our tool, the most strong argument here is the well prediction of the time course of the complex redistribution of ions caused by stopping the Na^+^/K^+^ pump using the parameters obtained from analysis of the balanced ion distribution. A full reverse of the intracellular K^+^/Na^+^ ratio and a strong cell depolarization take place in this case. One could suppose that properties of channels and transporters will change. However, calculations with invariant parameters of channels and transporters showed well matching of calculated and real data. One could suppose that the case of parameters alteration occurs more often than the parameters constancy. Of course, it would be nice to have another independent method for determining parameters, but it still needs to be looked for. Current study demonstrates using the computational tool for evaluation the parameter changing.

### Notes Added in Response to Some Readers of Our Preprint Posted at BioRchiv (Yurinskaya et al., 2018)

Some of our readers have expressed doubt that using our tool, one can get a unique set of parameters that provide agreement between experimental and calculated data. Therefore, a few comments would be in order.

The problem studied and discussed in our manuscript means mathematically a search for coefficients of a system of ordinary differential equations with non-linear right-hand sides. There is a vast area of applied mathematics dealing with similar problems using numerical methods (see e.g., Kahaner et al., 1989; Bonnans et al., 2006). It is known that the problem can be solved in a practically meaningful sense only if certain constraints on parameters and variables are accepted which are obtained from the knowledge of properties of a real object. Studying of our real object showed that the effects of the different parameters on the state of the system may vary widely in different areas. This indicates the poor “conditionality” of the so-called Hessian matrix. It is impossible to predict theoretically in our case how many sets of parameters will give one and the same results. However, the use of our executable file allows us to solve the problem by trial testing various parameters in the area of interest. The procedure in practice is not too time consuming. This is what we did in our previous (Vereninov et al., 2014, 2016) and current studies. Some obvious constraints are included directly in our source code. These are: *U* < 0, [Na]_i,o_ > 0, [K]_i,o_ > 0, [Cl]_i,o_ > 0, *V* > 0. The initial parameters in our treatment are found for the “balanced state of cell” (this common physiological term means that the inward and outward fluxes for each of ion species capable of crossing the cell membrane are equal). This means, mathematically, that only those systems are considered for which a stationary state is possible. If explicit constraints are not enough, it is the researcher himself who knows the real object should find additional constraints that allow finding a unique set of parameters or at least to limit the number of possible sets. In some cases, searching for certain parameters may be unnecessary because the executable file shows that the driving forces (*mun, muk, mucl*) are low or zero in the considered area. In other cases, using specific blockers of channels or transporters might help. A bit of creativity and inventiveness is required here. Our articles demonstrate how a problem was solved in some real cases. In the present study the ratio of ouabain-sensitive to ouabain-resistant components of the rubidium influx (OSOR) helps to choose the right set of parameters. OSOR is obtained from experimental data easily and reliably. Its value is included in the output table. Using our executable file helps to determine which parameter(s) most strongly affect cell ion homeostasis under given conditions and to find the optimal experimental protocol for studying its role in the considered phenomena.

## Conclusions

1. The experimental data on the time course of K^+^, Na^+^, and Cl^−^ concentrations and ouabain-sensitive and -resistant Rb^+^ influx in U937 cells treated with STS for 0.5–4 h enabled us to evaluate the changes in the pump rate coefficient and to compute alterations of the K^+^, Na^+^, and Cl^−^ channel permeability coefficients associated with the initial stages of apoptosis and AVD.
2. The redistribution of K^+^, Na^+^, and Cl^−^ underlying AVD in U937 cells is caused (1) by a progressive decrease in the Na^+^/K^+^ pump rate coefficient from an initial 0.029 to 0.013 at 4 h, (2) by a significant increase in pCl (0.013–0.068) and increases in pK (0.012–0.03, later 0.02), and (3) by a moderate decrease in pNa (0.004–0.003). The most critical factors for changes in cell K^+^ and Na^+^ are the suppression of the pump, an increase in pK and a decrease in pNa, whereas the early decrease in Cl^−^ and water content (early AVD) are associated primarily with an increase in pCl by ~5 times and an increase in pK by ~2.6 times.
3. Our approach demonstrates how to calculate the dependence of cell ion and water balance on the states of channels and transporters in the plasma membrane and is recommended for analyzing redistribution of monovalent ions and water not only during apoptosis but in other cases as well.

## Author Contributions

All authors contributed to the design of the experiments, performed the experiments, and analyzed the data. IV developed algorithm of numerical solution of the problem and wrote the programme code. AV wrote the manuscript with input from all authors. All authors have approved the final version of the manuscript and agree to be accountable for all aspects of the work. All persons designated as authors qualify for authorship, and all those who qualify for authorship are listed.

### Conflict of Interest Statement

The authors declare that the research was conducted in the absence of any commercial or financial relationships that could be construed as a potential conflict of interest.
